# Concordance between genomic alterations assessed by next-generation sequencing in tumor tissue or circulating cell-free DNA

**DOI:** 10.18632/oncotarget.11692

**Published:** 2016-08-30

**Authors:** Young Kwang Chae, Andrew A. Davis, Benedito A. Carneiro, Sunandana Chandra, Nisha Mohindra, Aparna Kalyan, Jason Kaplan, Maria Matsangou, Sachin Pai, Ricardo Costa, Borko Jovanovic, Massimo Cristofanilli, Leonidas C. Platanias, Francis J. Giles

**Affiliations:** ^1^ Developmental Therapeutics Program of Division of Hematology Oncology, Northwestern University, Chicago, IL, USA; ^2^ Department of Medicine, Northwestern University Feinberg School of Medicine, Chicago, IL, USA; ^3^ Robert H. Lurie Comprehensive Cancer Center of Northwestern University, Chicago, IL, USA; ^4^ Department of Medicine, Jesse Brown Veterans Affairs Medical Center, Chicago, IL, USA

**Keywords:** next-generation sequencing, cell-free DNA, genomic alterations, metastatic disease, lung cancer

## Abstract

Genomic analysis of tumor tissue is the standard technique for identifying DNA alterations in malignancies. Genomic analysis of circulating tumor cell-free DNA (cfDNA) represents a relatively non-invasive method of assessing genomic alterations using peripheral blood. We compared the concordance of genomic alterations between cfDNA and tissue biopsies in this retrospective study. Twenty-eight patients with advanced solid tumors with paired next-generation sequencing tissue and cfDNA biopsies were identified. Sixty-five genes were common to both assays. Concordance was defined as the presence or absence of the identical genomic alteration(s) in a single gene on both molecular platforms. Including all aberrations, the average number of alterations per patient for tissue and cfDNA analysis was 4.82 and 2.96, respectively. When eliminating alterations not detectable in the cfDNA assay, mean number of alterations for tissue and cfDNA was 3.21 and 2.96, respectively. Overall, concordance was 91.9–93.9%. However, the concordance rate decreased to 11.8–17.1% when considering only genes with reported genomic alterations in either assay. Over 50% of mutations detected in either technique were not detected using the other biopsy technique, indicating a potential complementary role of each assay. Across 5 genes (*TP53, EGFR, KRAS, APC, CDKN2A*), sensitivity and specificity were 59.1% and 94.8%, respectively. Potential explanations for the lack of concordance include differences in assay platform, spatial and temporal factors, tumor heterogeneity, interval treatment, subclones, and potential germline DNA contamination. These results highlight the importance of prospective studies to evaluate concordance of genomic findings between distinct platforms that ultimately may inform treatment decisions.

## INTRODUCTION

A central goal of precision medicine in oncology is to target genomic alterations with novel therapeutic agents in a timely manner as the tumor genomic profile evolves. Currently, genomic analysis of tissue biopsy is accepted as the gold standard strategy for identifying DNA genomic alterations in tumors using next-generation sequencing (NGS) among other techniques. While tumor tissue will continue to remain the optimal technique to guide targeted treatments and to best understand tumor architecture and histology, limitations exist. In some circumstances, tissue biopsies carry risks and can be technically challenging to repeat. For instance, major complication rates with thoracic biopsies have been reported at 5.2% [1]. In other instances, such as metastatic disease, actionable molecular alterations may exist at multiple sites, not easily accessible via a single tissue biopsy. Therefore, developing non-invasive techniques for characterizing tumor mutations at baseline and dynamically during treatment may be necessary to assess tumor evolution, to monitor therapy response, and to personalize changes in treatment.

Tumor heterogeneity represents a major challenge to personalized anticancer therapy. Intratumor heterogeneity can result in missing critical DNA genomic alterations using conventional tumor biopsies and underestimation of the genomic variability within a tumor [2–5]. While tumor tissue-based biopsies are limited by spatial and temporal (e.g., potential need for multiple invasive biopsies) considerations, circulating tumor cell-free DNA (cfDNA) assays have emerged as a less invasive method of assessing tumor genomic alterations using peripheral blood [6, 7]. cfDNA quantity is on average higher in patients with cancer compared to controls, but varies considerably, and is thought to arise from apoptotic and necrotic cells [8]. In localized disease, the proportion of purified cfDNA in the blood is extremely low, which may limit utility in these patients. For advanced tumors, cfDNA is variable with some tumor types such as pancreatic, ovarian, colorectal, breast, bladder, esophageal, melanoma, and hepatocellular carcinoma expressing higher percentages of cfDNA while others, such as brain, renal, prostate, and thyroid cancers having detectable circulating DNA in less than 50% of patients [9]. One study with an estimated 95% of patients having advanced or metastatic disease reported 58% of patients having at least one detectable alteration, which increased to 65% when excluding glioblastoma [10]. In contrast, only 1 of 222 (0.45%) healthy controls was found to have an alteration present. In addition, tumor purity based on non-cancerous cells in the tumor microenvironment may also complicate cfDNA assays [11]. Still, cfDNA has the potential to capture DNA alterations in the peripheral blood in a more dynamic manner in particular types of advanced tumors when feasibility of repeat tissue biopsies is limited.

Potential applications of cfDNA assays include early detection of metastatic disease and monitoring of minimum residual disease [12]. For example, detection of emerging *EGFR* mutations (e.g., deletions in exon 19 and L858R substitutions in exon 21) in patients with non-small cell lung cancer (NSCLC), can guide treatment with EGFR tyrosine kinase inhibitors (TKIs) [13, 14]. In many cases, tumor evolution results in secondary *EGFR* T790M mutations in exon 20 leading to resistance to EGFR TKIs [15]. cfDNA assays may have the potential to identify when these secondary resistance mutations arise in the peripheral blood prior to detection of clinical or radiological progression of disease. A recent prospective study indicates potential for detecting *EGFR* and *KRAS* mutations with 100% positive predictive value using plasma droplet digital PCR, which can be used to detect a small number of known mutation targets [16]. *EGFR* T790M mutations were also examined with specificity of 63%, possibly related to tumor heterogeneity and false-negative tissue genotyping. In addition, recent studies have suggested that detection of mutational burden can potentially help predict response to immunotherapies such as the checkpoint inhibitors targeting programmed death 1 (PD-1) and programmed death ligand 1 (PD-L1), raising another potential application of cfDNA analysis in parallel with genomic analysis of tissue biopsies [17–19].

High concordance has been reported between tumor tissue NGS and cfDNA in studies investigating the presence of *EGFR* alterations in NSCLC, multiple genes in pancreaticobiliary cancers (*KRAS*, *TP53*, *APC*, *FBXW7*, *SMAD4*), exons 12–13 of *KRAS* in colorectal cancer, *BRAF* V600E and *KIT* mutations in melanoma, and *BRAF*, *EGFR*, *KRAS*, and *PIK3CA* across a variety of advanced cancers [20–23]. These studies report high specificity and diagnostic accuracy as greater than 80–90% compared to the gold standard of tissue-based NGS. However, in these studies, the reported values are based predominantly on not detecting DNA alterations in either assay (e.g., no mutations detected in *EGFR* in the same patient). This limits potential applicability for whether this technology can be used to detect early mutations in the peripheral blood. One study reported an average concordance of 85.9% in advanced cancers when including mutations that were both present and absent and 90% when limiting the sample to patients with stage II colorectal cancer [22]. Other work has reported high concordance for real-time polymerase methods to detect targeted *BRAF* V600 mutations with rapid turn around time [24].

In early-stage disease, the low levels of cfDNA in peripheral blood may limit long-term clinical applications. In advanced cancers, current data are lacking that targeting cfDNA mutations in the peripheral blood improve patient outcomes. More clinical data are necessary to determine whether NGS data derived from cfDNA assays sufficiently correlate with that obtained from tissue biopsies to determine if and when cfDNA assays may be beneficial clinically. The utility may exist in detecting treatment response and resistance, as opposed to replacing tumor biopsy for initial treatment decision making [25].

The goal of the present study was to identify concordance of genomic alterations obtained from tissue biopsies and cfDNA analyses for patients with advanced malignancy. This is necessary in order to assess the fidelity of cfDNA as many genomic alterations contribute to tumor heterogeneity. It is also critical to understand whether this information may be useful in patients in whom tumor tissue is unavailable to support clinical treatment decisions based on emergence of genomic alterations that can predict resistance to treatment. To our knowledge, this is one of the most systematic analyses in terms of number of genes to examine concordance across DNA alterations as assessed in tissue-based NGS and cfDNA.

## RESULTS

### Patient characteristics

Fifty-four patients were identified retrospectively to have cfDNA testing performed by a single commercial NGS sequencing provider. Of these, 29 patients had matched tumor tissue biopsy and peripheral blood cfDNA genomic analyses. One patient was excluded for insufficient sample for tissue-based FoundationOne testing. Table 1 shows the patient characteristics of the 28 patients included in the study. There were 14 lung cancer (10 adenocarcinoma, 1 poorly differentiated NSCLC, 1 squamous, 1 small cell, 1 large cell neuroendocrine), 3 ovarian (2 serous, 1 clear cell), 2 endometrial (1 mucinous, 1 epitheloid), 2 thyroid (1 papillary, 1 poorly differentiated not otherwise specified), 2 hepatocellular (1 clear cell, 1 undifferentiated), 2 unknown primary, 1 cholangiocarcinoma (mucinous), 1 gastroesophageal junction adenocarcinoma, and 1 peritoneal adenocarcinoma (serous). Overall, 14 of 28 (50%) patients had lung cancer and 26 of 28 (93%) had stage IV disease. The median interval between collecting each paired tumor biopsy was 89 days [8–3,448 days]. Nine patients had no treatment between biopsy collections.

**Table 1:** Characteristics of patients with both tissue and cell-free DNA NGS testing

	Number	Percentage (%)
**Age (years)**		
Median	65	
**Sex**		
Male	9	32.1
Female	19	67.9
**Type of cancer**		
Lung	14	50.0
Ovarian	3	10.7
Endometrial	2	7.1
Thyroid	2	7.1
Hepatocellular	2	7.1
Unknown primary	2	7.1
Cholangiocarcinoma	1	3.6
Gastroesophageal junction	1	3.6
Peritoneal carcinoma	1	3.6
**Pathologic stage**		
III	2	7.1
IV	26	92.9
**History of prior cancers**	7	25.0
**Smoking History**		
Current/Former	16	57.1
Never	12	42.9
**Biopsy site corresponds to primary tumor**		
Yes	12	42.9
No	14	50.0
Unknown	2	7.1
**Interval between tissue and blood sample collection**		
< 90 days	14	50.0
> 90 days	14	50.0

### Concordance of tumor biopsy and cfDNA genomic analyses

Concordance between the two assays was 91.9% (1672/1820 genes) including all genes examined (SD 4.31%) (Table 2). Concordance was similar for the 14 lung cancer patients (91.1%) as compared to the 14 patients with non-lung cancers (92.6%). When excluding particular alterations within overlapping genes not sequenced by Guardant360, concordance was 93.9%. For this analysis, concordance was also similar when comparing lung cancer patients (93.3%) to non-lung cancer patients (94.5%). Concordance was high across all patients with range of 81.5%–100%. One of 28 (3.6%) had complete concordance at the patient level. This patient had no genomic alterations detected in either assay.

**Table 2:** Composite NGS data comparing tissue biopsy with cfDNA

Average concordance of genomic analyses when DNA alterations are present or absent	91.9%	93.9%^+^
Percent of tissue alterations found in cfDNA	20.7%	31.1%^+^
Percent of cfDNA alterations found in tissue	33.7%	33.7%^+^
Mean and SD of genomic alterations in cfDNA	2.96 (3.01)	2.96 (3.01)^+^
Mean and SD of genomic alterations in tissue	4.82 (3.02)	3.21 (2.25)^+^

Note: variants of unknown significance (VUS) included; cfDNA, cell-free DNA; SD, standard deviation.

+ excludes variants within overlapping genes not tested by Guardant360.

The remaining analyses were subset analyses, specifically examining concordance for genes with a genomic alteration present in one or both assays (i.e., excluding double negatives, wild type/wild type). Among the subset of genes with reported genomic alterations in either assay (*N* = 170), concordance between the two assays was 11.8% with a partial concordance of 4.7%. The full and partial concordance values were 17.1% and 4.7%, respectively, when only including alterations detectable in both assays. When excluding cfDNA biopsies without any alterations detected (*N* = 6), concordance and partial concordance were 19.0% and 5.2%, respectively. No significant differences were found when stratifying the sample based on genes with complete exon sequencing or copy number variant (CNV). The gene level concordance for each individual patient ranged from 0 to 33.3% with partial concordance of 0 to 28.6%. When only examining variants detectable in both assays, gene level concordance was 0 to 66.7% with partial concordance of 0 to 28.6%. The sample was also analyzed based on collection interval between biopsies, less than 90 days versus greater than 90 days (Table 3). Concordance was 12.7% with a partial concordance of 1.3% for results of both tumor tissue and cfDNA less than 90 days apart (*N* = 77). For biopsies more than 90 days apart, concordance was 10.8% with a partial concordance of 7.5% (*N* = 93). The trend was similar when only including alterations detectable in both assays. In addition, when examining concordance for patients with no treatment between biopsies (*N* = 9), no significant differences were noted in this preliminary analysis.

**Table 3:** Concordance and partial concordance among only genes with genomic alterations in either assay

	Genes with DNA aberrations (*N* = 170)	Genes with DNA aberrations+ (*N* = 129)	< 90 days between biopsies (*N* = 77)	> 90 days between biopsies (*N* = 93)	< 90 days between biopsies+ (*N* = 58)	> 90 days between biopsies+ (*N* = 71)
Concordance	11.8%	17.1%	12.7%	10.8%	17.2%	16.8%
Partial Concordance	4.7%	4.7%	1.3%	7.5%	1.7%	7.0%

*N* = number of unique genes with DNA alterations.

Note: variants of unknown significance (VUS) included.

+ excludes variants within overlapping genes not tested by Guardant360.

**Figure 1:**
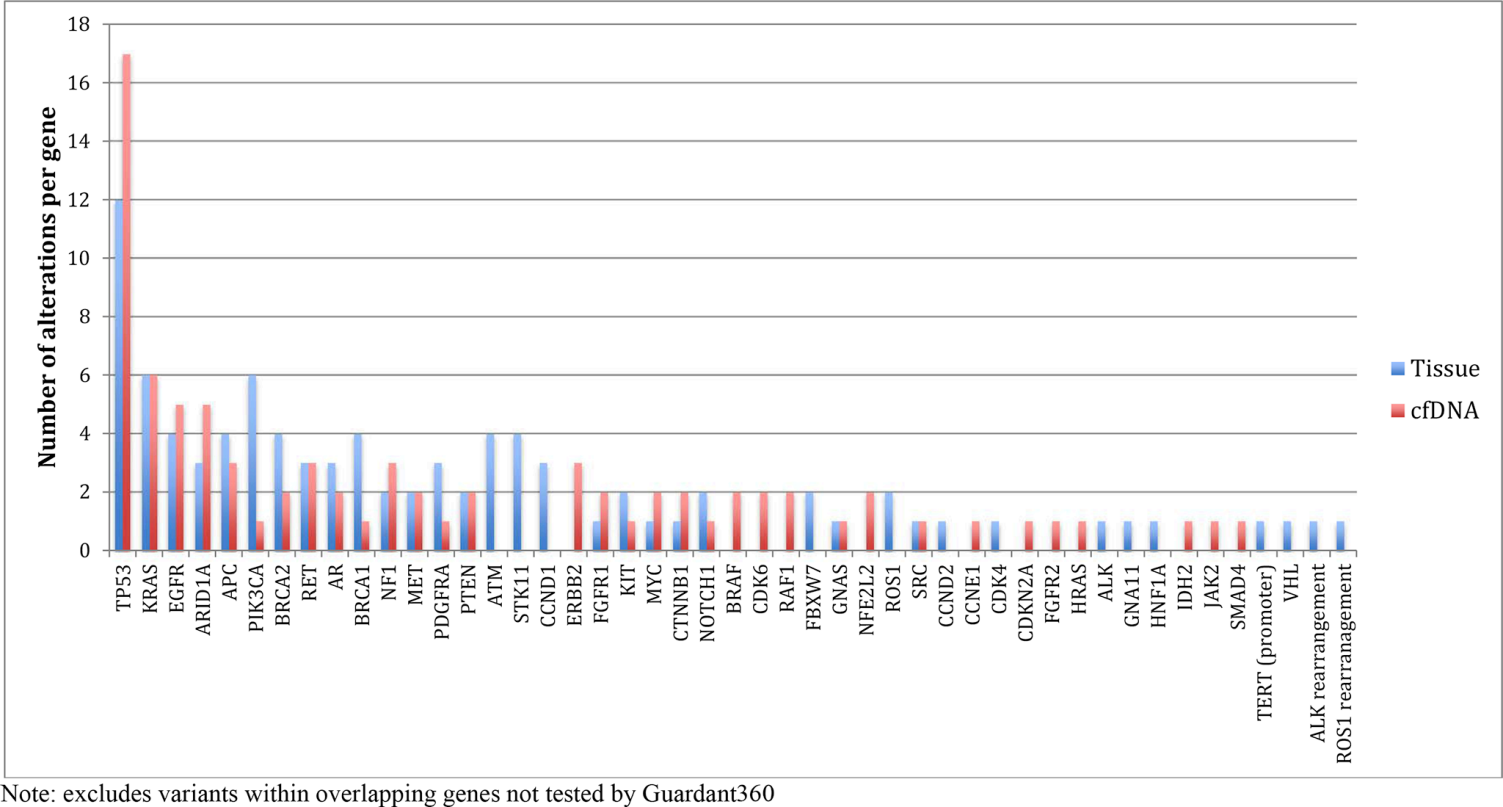
Comparing frequency of alterations per gene in tissue and plasma cfDNA

**Figure 2:**
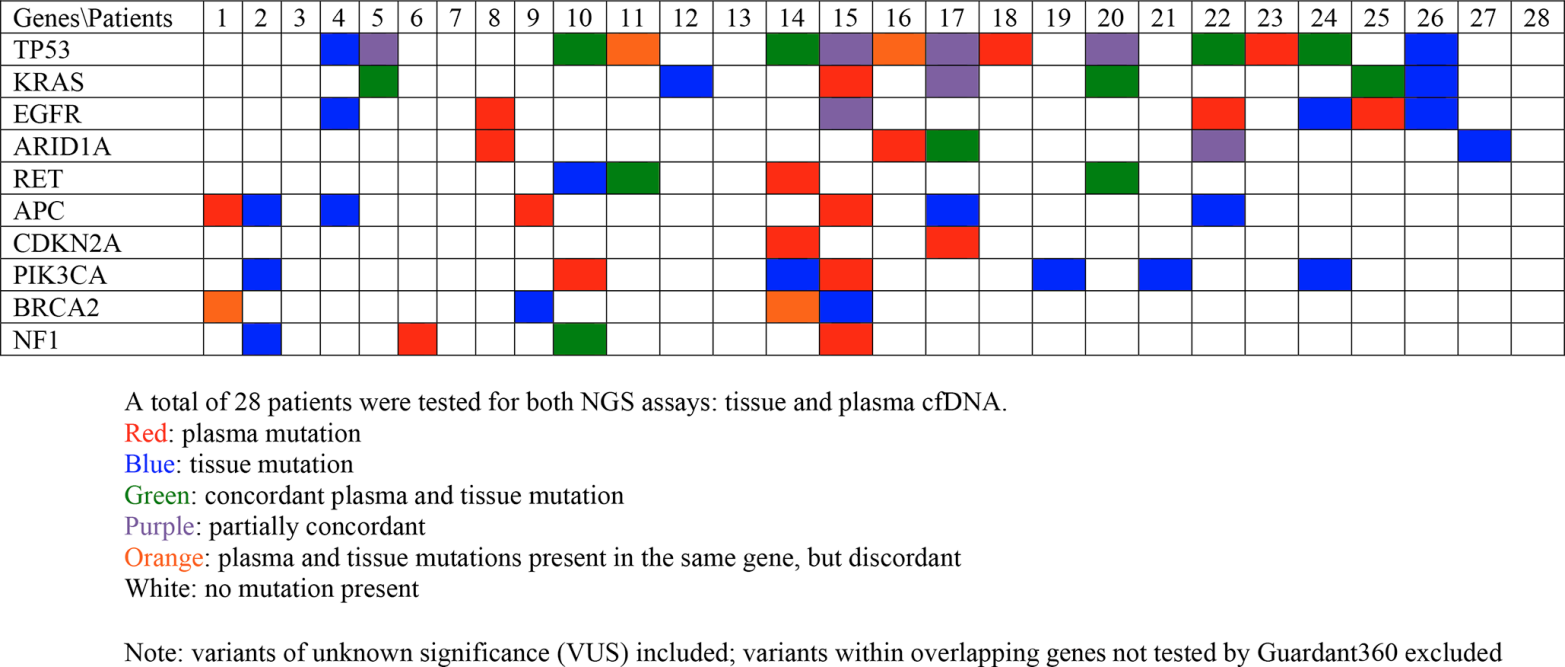
Oncoprint chart for 10 representative genes across all patients

When considering the same genes and variants analyzed by both platforms, 31.1% of tissue-based mutations were detected via cfDNA. In addition, 33.7% of cfDNA mutations were detected via tissue-based sequencing. Figure 1 shows the landscape of DNA mutations found in the NGS platforms in each patient. Figure 2 is an oncoprint chart displaying the different potential outcomes observed for the 10 representative genes across all patients.

### Cell-free DNA percent

For genes with identical sequencing mutations (*N* = 23), the percent or allele frequency of altered cfDNA was analyzed. The mean allele frequency of altered cfDNA in the peripheral blood for the subset of mutations found in both tissue and circulating blood was 16.8% (SD 27.4%) [range 0.1%–81.0%]. Overall, 65% of these identical mutations (15 of 23), were found to have relatively low (less than 10%) allele frequency of altered circulating cfDNA.

### Average number of DNA alterations

The average number of alterations including VUS per patient for tissue and cfDNA was 4.82 (SD 3.02) and 2.96 (SD 3.01), respectively (Table 2). Median number of mutations for tissue and cfDNA was 4 and 2, respectively. More mutations were detected in tissue-based NGS in 21 of 28 (75%) of patients. A greater number of genomic alterations were detected in cfDNA in 5 of 28 (17.9%) patients. An equal number of mutations were detected in 2 of 28 (7.1%) patients. When excluding particular alterations within overlapping genes not sequenced by Guardant360, average number of alterations including VUS for tissue and cfDNA was 3.21 (SD 2.25) and 2.96 (SD 3.01). Median number of alterations was 3 for the tissue assay and 2 for the cfDNA assay.

### Diagnostic accuracy analysis

Gene-level sensitivity, specificity, positive predictive value (PPV), negative predictive value (NPV) and diagnostic accuracy were analyzed across the five representative genes in the sample - *TP53*, *EGFR*, *KRAS*, *APC*, *CDKN2A* (Table 4). Tissue-based NGS was used as the gold standard for this analysis [26]. Across all 5 genes examined, including VUS, sensitivity was 50.0%, specificity was 89.5%, and diagnostic accuracy was 82.6% (Supplementary Table S3). Values were slightly higher when VUS were excluded with sensitivity of 59.1%, specificity of 94.8%, and diagnostic accuracy of 89.1% (Table 4). Four of the 5 genes examined had a specificity of greater than 90%. Specificity and diagnostic accuracy were lowest for TP53. The Youden's J index across all 5 genes was 0.4 when including VUS and 0.5 when excluding VUS.

**Table 4:** Sensitivity, specificity, and diagnostic accuracy across 5 genes

		Tissue Mutations		Sensitivity (%)	Specificity (%)	PPV (%)	NPV (%)	Diagnostic Accuracy (%)	Youden's J index
cfDNA mutations		(+)	(−)						
TP53	(+)	8	2						
	(−)	2	14	80.0	87.5	80.0	87.5	78.6	0.7
EGFR	(+)	1	2						
	(−)	1	24	50.0	92.3	33.3	96.0	89.3	0.4
KRAS	(+)	4	1						
	(−)	2	21	66.7	95.5	80.0	91.3	89.3	0.6
APC	(+)	0	1						
	(−)	4	23	0.0	95.8	0.0	85.2	82.1	0
CDKN2A	(+)	0	0						
	(−)	0	28	n/a	100.0	n/a	100.0	100.0	n/a
Total positive		13	6						
Total negative		9	110						
Total (positive + negative)		22	116	59.1	94.8	68.4	92.4	89.1	0.5

PPV: positive predictive value

NPV: negative predictive value

cfDNA: cell-free DNA

Note: variants of unknown significance (VUS) and variants not tested by Guardant360 excluded.

## DISCUSSION

Personalized treatment in oncology aims to treat malignancies based on their genomic profile with effective molecularly targeted drugs. Additionally, the goal is to detect the emergence of genetic alterations that predict recurrence or resistance to treatment prior to development of clinical symptoms or radiological evidence of disease progression. Rapid developments in genomic analyses of tumors are enabling this transformative paradigm in oncology that may ultimately allow real-time treatment decisions based on the precise genomic landscape of the tumor. In certain circumstances, genomic analysis of cfDNA may hold promise to help overcome this challenge if findings reliably correlate with primary tumor and metastatic site(s) genomic landscape. Hence, the primary objective of this study was to investigate the concordance rate of genomic profiling using NGS in both tissue biopsies and peripheral blood circulating tumor cfDNA.

As compared to tissue-based biopsy, our findings indicate that cfDNA assays have high specificity, but low sensitivity, along with diagnostic accuracy in the range of 82–89%. When examining all genes, including those without DNA alterations in either assay, concordance was 91.9–93.9%. However, when examining the subset of genes with DNA alterations found in either assay, concordance and partial concordance were low (11.8–17.1% and 4.7%, respectively). A recent study examining 50 hotspot genes in tumor and cfDNA reported a sensitivity of 49.9% and a specificity of 99.8% for patients with advanced or metastatic solid tumors [27]. Similarly, previous studies that report high concordance and specificity are based on single genes and sometimes specific codons. A critical difference in our analyses was that the genomic platforms used in our study included genes with both critical and complete exon coverage, thereby looking more comprehensively at sequencing concordance of the 65 overlapping genes. This analysis was critical to appropriately assess the potential for cfDNA to accurately identify specific sequencing mutations and resistance patterns. For example, it is not sufficient to classify a *BRAF* V600E mutation in tissue and a *BRAF* S729L VUS in peripheral blood as concordant at the gene level. Instead, we only considered concordant DNA alterations when the exact same sequencing alteration was present in both biopsies. Collectively, these data indicate that cfDNA assays may be best utilized to rule in rather than to rule out certain genomic alterations given the high specificity.

There are several potential reasons to explain the difference in results between the two NGS assays. First, the biopsy techniques are quite different. In lung cancer, previous studies using multi-region whole-exome and/or whole-genome sequencing indicate differing degrees of driver mutation heterogeneity with one study reporting 20/21 known cancer gene mutations in all regions [28]. However, another study suggested that subclonal diversification results in missed driver mutations indicating that multiple tissue biopsies in different locations would be optimal to best characterize tumor heterogeneity [29]. In contrast, cfDNA only captures mutations above the detection threshold that are found in peripheral blood after tumor cells outgrow the blood supply, become hypoxic, and undergo apoptosis or necrosis, releasing DNA into the peripheral blood. Therefore, there are intrinsic differences in assay platform and sensitivity. With current sequencing technology, more genomic alterations were detected in tissue biopsies (mean 4.82) as compared to cfDNA (mean 2.96). However, when eliminating alterations not detectable with the cfDNA at this time, mean alterations for tissue (3.21) and cfDNA (2.96) were similar. While our analyses compared a single, commercially available cfDNA assay to tissue-based NGS, the emergence of additional cfDNA platforms necessitates further studies comparing these to tissue-based NGS. Studies are ongoing to assess concordance using new platforms, as well as more sensitive droplet digital PCR for targeted sequencing alterations.

Second, temporal factors may also be significant. Tumors are highly dynamic and a larger sample size stratifying concordance based on timeframe between biopsies is critical. For patients with advanced cancer and sufficient cfDNA in the blood, we hypothesized that higher concordance would be associated with closer timeframe between biopsies. We were unable to validate this hypothesis based on our analysis (Table 3).

Third, type of cancer and site of biopsy are likely important. Some types of tumors, such as pancreatobiliary cancers, are more difficult to biopsy and to capture heterogeneity with tissue-based biopsies. Previous studies have also indicated differences in regard to type of malignancy and degree of cfDNA detected in the blood. Pancreatic, ovarian, colorectal, breast, bladder, gastroesophageal, melanoma, and hepatocellular carcinoma were more likely to have detectable cfDNA as compared to primary brain, renal, prostate, and thyroid cancers [9]. Our study indicated that concordance for patients with lung cancer was similar when compared to concordance for non-lung cancers. In addition, in our sample, 50% of tissue biopsies were performed at metastatic sites, which likely captures greater tumor heterogeneity after accumulation of mutations that promote metastasis. The burden and location of metastatic disease also may play a role in cfDNA detection and genomic landscape heterogeneity.

Finally, we included both subclones and VUS in this analysis with the goal of fully analyzing sequencing concordance between the two techniques. However, the potential to make treatment related decisions based on these assays is mostly predicated on clinically significant mutations at this time. Further comprehensive preclinical studies are needed to analyze this subset of mutations to determine the true functional significance of such variants. In addition, future studies should examine concordance comparing synonymous mutations across NGS platforms. Interestingly, over half of mutations detected in either technique were not detected using the other biopsy technique (Table 2, Supplementary Table S4). This finding indicates considerable tumor heterogeneity that cannot be fully detected in either biopsy technique alone. This implies a potential complementary role of both tissue-based and cfDNA biopsies to better capture tumor heterogeneity.

To the best of our knowledge, the study examines the concordance of the largest number of genes (65) to date and reflects what is found in a real-world oncology clinical setting. Our analyses were comprehensive in assessing concordance for the panel of overlapping genes tested in both NGS platforms: tissue and blood cfDNA. We defined concordance in our study to encompass the full set of genes tested, rather than focusing on each gene. We included VUS and subclones to fully capture sequencing concordance. Theoretically, there should be no difference in concordance between VUS and non-VUS. This is supported by similarities in our sensitivity, specificity, and diagnostic accuracy analyses with and without VUS (Table 4 and Supplementary Table S3). We included a heterogeneous group of cancers in our analysis to optimally examine concordance across multiple types of cancer. Limitations of our study include a relatively small patient sample size, and half of the sample consisting of patients with lung cancers, which may have skewed the most common genes encountered in our sample. A large prospective trial assessing concordance between circulating tumor DNA and FoundationOne matched solid tumor samples is currently underway (NCT02620527).

In conclusion, our findings indicate high specificity and concordance when genomic alterations are present or absent. When examining the subset of genes with DNA alterations present, concordance was relatively low. Further studies are warranted to validate our findings across multiple cancer types, to examine concordance as new cfDNA platforms and sequencing technologies develop, to compare concordance at different intervals between biopsies, and to determine change in cfDNA genomic alteration type and frequency over time.

## MATERIALS AND METHODS

### Study design and patients

The Institutional Review Board of Northwestern University approved the study. All patients were recruited within the Northwestern Medicine Developmental Therapeutics Program, Division of Hematology Oncology, Northwestern University Feinberg School of Medicine. All studies were conducted in concordance with the Declaration of Helsinki. Fifty-four consecutive patients with commercial cfDNA NGS testing by Guardant360 (Guardant Health, Redwood City, CA) were identified retrospectively. Of these, 29 patients had comprehensive tissue NGS testing (FoundationOne) commercially performed by Foundation Medicine (Cambridge, MA) [26]. One patient was excluded because the FoundationOne report stated inadequate sample quality. Therefore, the final sample size was 28 patients with both tissue and peripheral blood cfDNA results available. Clinical characteristics of patients included in the study were retrospectively obtained via patient chart review. These data included basic demographics as well as tumor biopsy information to characterize the histology and stage of malignancy.

### Genes analyzed

The study examined concordance between all genes found in cfDNA that were also present in tumor biopsy samples. In total, 68 genes were tested by the Guardant360 cfDNA. Of those, 3 genes were excluded (*RHEB*, *RHOA*, and *RIT1*) because these genes were not included in the 315 gene panel tested by tissue biopsy sequencing. Therefore, 65 genes common to both assays were examined for concordance (Supplementary Table S1). Twenty-nine genes had complete exon sequencing by both assays. Thirty-nine genes had critical exon sequencing by Guardant360 and complete exon sequencing by Foundation Medicine. Four rearrangements were common to both assays. The median interval between collections of tissue biopsy and peripheral blood specimens was 89 days.

### Defining concordance and data analysis

Concordance analysis between genomic findings from tumor tissue biopsy and plasma cell-free DNA was performed on 65 genes. Two definitions of concordance were utilized in the study. First, concordance was defined at the gene level as detecting an identical sequencing mutation or not detecting an alteration in a single gene. For example, a R248L mutation in *TP53* detected in both assays for the same patient was counted as a concordant genetic alteration. In contrast, the finding of distinct mutations detected in the same gene when the two assays were performed on the same patient was counted as a discordant genomic alteration (e.g., *TP53* R248W by cfDNA and *TP53* K132R by tissue biopsy) (Supplementary Table S2). For this analysis, the denominator in this calculation was 1,820 (65 genes for 28 patients).

Second, concordance was examined for the subset of genes in which a genomic alteration was found. For this analysis, genes in which mutations were not found (e.g., no mutations found in *EGFR* in both assays in the same patient) were excluded from both the numerator and denominator. Concordance was further compared when excluding particular alterations within overlapping genes not sequencing by Guardant360. These included splice site mutations, certain small insertions or deletions, and allelic loss (such as *PTEN*). Partial concordance was defined as having one concordant mutation and at least one discordant genomic alteration in the same gene. Alterations were binned into 3 categories: concordant, partially concordant, or discordant. Total concordance was defined, not by patients, but by the total number of fully concordant or partially concordant alterations with the denominator as the total number of DNA alterations in our sample (*N* = 170 genomic alterations or *N* = 129 when excluding alterations not sequenced by the cfDNA assay). The analysis included non-synonymous DNA mutations, rearrangements, and copy number variants (CNV) regardless of clone percentage. As variants of unknown significance (VUS) are also important to assess sequencing concordance, these were also included. All cfDNA samples, regardless of the number of DNA alterations detected (e.g., even when none were detected) were included. Synonymous DNA alterations reported by Guardant360 were not included in any concordance analysis because synonymous alterations were not included in FoundationOne reports.

In addition, sensitivity, specificity, and diagnostic accuracy (effectiveness) analyses were performed across the 5 representative genomic alterations (*TP53*, *EGFR*, *KRAS*, *APC*, *CDKN2A*) in the sample (Figure 1). This analysis included instances in which no alterations were detected in either assay (double negatives). There were two instances for *TP53* in which a DNA alteration was detected in the same gene in both assays, but the sequencing mutation was discordant. These two samples were excluded, only from the sensitivity, specificity, and diagnostic accuracy analysis for *TP53* (*N* = 26). All other analyses included 28 patients. Youden's J index (sensitivity + specificity – 1) was calculated as an indirect measurement of concordance, as well as an alternative method reflecting diagnostic accuracy [30].

## SUPPLEMENTARY MATERIALS TABLES


