# Supplementary material for: Concordance between genomic alterations assessed by next-generation sequencing in tumor tissue or circulating cell-free DNA
Source: Oncotarget. 2016 Aug 30;7(40):65364–73. doi: 10.18632/oncotarget.11692 (PMC5323161; doi:10.18632/oncotarget.11692)
Supplement: Supplementary file 1 [file oncotarget-07-65364-s001.pdf]

Concordance between genomic alterations assessed by next-generation sequencing in tumor tissue or circulating cell-free DNA

Supplementary Materials

Supplementary Table S1: Genes and rearrangements studied

Genes with complete exon and partial intron coverage by Guardant360	*APC, AR, ARD1A, BRAF, BRCA1, BRCA2, CCND1, CCND2, CCNE1, CDK4, CDK6, CDKN2A, CDKN2B, EGFR, ERBB2, FGFR1, FGFR2, HRAS, KIT, KRAS, MET, MYC, NF1, NRAS, PDGFRA, PIK3CA, PTEN, RAF1, TP53*
Genes with critical exon coverage by Guardant360	*AKT1, ALK, ARAF, ATM, CDH1, CTNNB1, ESR1, EZH2, FBXW7, FGFR3, GATA3, GNA11, GNAQ, GNAS, HNF1A, IDH1, IDH2, JAK2, JAK3, MAP2K1, MAP2K2, MLH1, MPL, NFE2L2, NOTCH1, NPM1, NTRK1, PTPN11, RET, ROS1, SMAD4, SMO, SRC, STK11, TERT, VHL*
Rearrangements	*ALK, NTRK1, RET, ROS1*

Note: *RHEB, RHOA, RIT1* were excluded from analysis as these genes were tested by Guardant360, but not by FoundationOne

Supplementary Table S2: Defining concordance and partial concordance

	Tissue *TP53*	cfDNA *TP53*
Concordant	*A*	*A*
Partially concordant	*A, B*	*A*
Discordant	*A*	*C*

Note: *A–C* refer to genomic sequencing alterations in a single gene for an individual patient

Supplementary Table S3: Sensitivity, specificity, and diagnostic accuracy across 5 genes, including variants of unknown significance (VUS)

		Tissue Mutations		Sensitivity (%)	Specificity (%)	PPV (%)	NPV (%)	Diagnostic Accuracy (%)	Youden's J index
**cfDNA mutations**		**(+)**	**(-)**						
TP53	(+)	8	2						
	(−)	2	14	80.0	87.5	80.0	87.5	78.6	0.7
EGFR	(+)	1	3						
	(−)	4	20	20.0	87.0	25.0	83.3	75.0	0.1
KRAS	(+)	3	2						
	(−)	2	21	60.0	91.3	60.0	91.3	85.7	0.5
APC	(+)	0	3						
	(−)	4	21	0.0	87.5	0.0	84.0	75.0	−0.1
CDKN2A	(+)	0	2						
	(−)	0	26	n/a	92.9	0.0	100.0	92.9	n/a
Total positive		12	12						
Total negative		12	102						
Total (positive + negative)		24	114	50.0	89.5	50.0	89.5	82.6	0.4

PPV: positive predictive value.
NPV: negative predictive value.
cfDNA: cell-free DNA.
Note: variants not tested by Guardant360 excluded.

Supplementary Table S4: Known or potentially actionable sequencing alterations detected in FoundationOne and Guardant360 across 65 overlapping genes

Patient Number	Tissue	cfDNA
1	**_GNAS_ amp-eq,** _SRC_ Q531*	_APC Q901*, SRC Q531*_
2	_APC_ E1540*, _PIK3CA_ W11L	None
3	None	None
4	_EGFR_ T751_L760 > NL, _TP53_ Y220C, _APC_ R213*	None
5	_CCND1_ amp, _CCND2_ amp, **_CDKN2A/B_ loss,** _FGFR1_ amp, _KRAS_ amp, _MYC_ amp, _TP53_ H179L, **_TP53_ L264fs*81**	_FGFR1_ amp, _KRAS_ amp, _MYC_ amp, _TP53_ H179L
6	_CTNNB1_ S45A	_JAK2_ V617F, _IDH2_ R140Q, _CTNNB1_ S45A
7	**_TP53_ V157fs*23**	None
8	**_TP53_ splice site 994-1G > A**	_ARID1A_ Q1250*, _CDK6_ amp
9	_BRCA2_ K944*, _CCND1_ amp, _CDK4_ amp-eq, **_TP53_ P27fs*17**	None
10	_NF1_ Y2285*, _TP53_ R267P, +	_TP53_ R267P, _BRCA1_ K1702E, _NF1_ Y2285*, _MET_ amp, _MYC_ amp, _BRAF_ amp, _PIK3CA_ amp, _RAF1_ amp, _CDK6_ amp
11	_RETCCDC6-RET_ fusion, _TP53_ H193L, _TERT_ promoter-146C > T	_RET_CCDC6-_RET_ fusion, _TP53_ E180*
12	_KRAS_ G12D, _GNAS_ R201H, **_TP53_ E294fs*52**	_GNAS_ R201H
13	_EGFR_ amp, **_EGFR-RAD51_ fusion**, **_ARID1A_ P1484fs*10**, _CCND1_ amp, **_CDKN2A/B_ loss**, **_JAK2_ amp-eq**	None
14	**_CDKN2A_ p16INK4a S8fs*7**, _PIK3CA_ amp-eq, _PTEN Y_180*, _TP53_ V272M	_TP53_ V272M, _PTEN_ Y180*
15	_EGFR_ amp, _TP53_ R248L	_TP53_ R248L, _TP53_ D41E, _CTNNB1_ S37C, _EGFR_ amp, _KRAS_ amp, _PIK3CA_ amp
16	_TP53_ K132R, **_ESR1_ amp**	_HRAS_ E31K, _TP53_ R248W, _TP53_ R273C, _TP53_ R110L
17	_APC_ I1307K, _KIT_ amp-eq, _KRAS_ G12C, _PDGFRA_ amp-eq, _TP53_ V157F, _STK11_ Q112*	_KRAS_ G12C, _KRAS_ amp, _TP53_ V157F
18	None	_TP53_ V274F
19	**_NF1_ loss**, **_TP53_ splice site 375G > T**	_RAF1_ S257L
20	**_CDKN2A/B_ loss**, _KRAS_ G12C, _TP53_ Y236C, _ATM Q2593*_	_KRAS_ G12C, _TP53_ Y236C, _TP53_ H179R
21	_FBXW7_ R505C, _PIK3CA_ E542K	None
22	**_CDKN2A/B_ loss**, _TP53_ R175H, _APC_ I1307K, _ARID1A_ W337*	_ARID1A_ R1989*, _ARID1A_ W337*, _TP53_ R175H, _EGFR_ amp
23	_ALK_ rearrangement intron 19	_TP53_ R175H
24	_TP53_ G199E	_TP53_ G199E
25	_KRAS_ G12F, _STK11_ K84*	_KRAS_ G12F, _EGFR_ C307*
26	_KRAS_ G12D, **_STK11_ splice site 863-1G > T**, _TP53_ R249S	None
27	_ARID1A_ Q605*, **_CDKN2A_ p16INK4a S12***, **_TP53_ splice site 783-2A > T**, **_STK11_ P281fs*6**	None
28	None	None

amp: amplification.
eq: equivocal.
+: FOne detected _BRCA1_ K1702E but categorized as VUS at the time.
Bolded variants: alterations within overlapping genes not sequenced by Guardant360.
